# COVID-19 home isolation and food consumption patterns: Investigating the correlates of poor dietary diversity in Lebanon: a cross-sectional study

**DOI:** 10.12688/f1000research.75761.1

**Published:** 2022-01-28

**Authors:** Maha Hoteit, Hussein Mortada, Ayoub Al-Jawaldeh, Carla Ibrahim, Rania Mansour

**Affiliations:** 1 Faculty of Public Health, Lebanese University, Beirut, Lebanon; 2 PHENOL research group (Public HEalth Nutrition prOgram-Lebanon), Faculty of Public Health, Lebanese University, Beirut, Lebanon; 3 Lebanese University Nutrition Surveillance Center, Lebanese University, Beirut, Lebanon; 4 Faculty of Science IV, Lebanese University, Beirut, Lebanon; 5 World Health Organization Regional Office for the Eastern Mediterranean, World Health Organization, Cairo, Egypt; 6 Department of Nutrition and food sciences, faculty of arts and sciences, Holy Spirit University of Kaslik, Jounieh, Lebanon; 7 Social Work Program, The Doha Institute for Graduate Studies, Doha, Qatar

**Keywords:** COVID-19, lockdown, Lebanon, food consumption pattern, food security, food consumption score, mitigation measures

## Abstract

**Background**: The unfurling COVID-19 pandemic has uncovered the defenselessness of the Lebanese food system leading to serious implication in maintaining a healthy sustainable lifestyle.

**Aim**: The main purpose of this study is to examine the impact of the COVID-19 pandemic on food consumption patterns and dietary diversity of the Lebanese people.

**Methods:** The online survey, completed between April and June 2020, consisted of a cross-sectional study on 2282 Lebanese participants (mean age: 29.36±12.221, 80.9% women) that was part of a survey across 38 different countries conducted by De Backer, C. *et al*. A food frequency questionnaire was used to investigate the consumption patterns along with the calculation of the Food Consumption Score (FCS), a proxy indicator of dietary diversity. Data collected on cooking attitudes, shopping, and food stock identify the community mitigation measures.

**Results**: Home isolation due to COVID-19 induced an increase in the consumption of legumes and pulses (3.2%, p-value=0.001) and whole wheat groups (2.8%, p-value=0.03). In contrast, a decrease of 5.4%, 6.9%, 5.8%, 5.1%, 3.1%, 3.4% and 2.8% was observed in the consumption of fruits (p-value=0), vegetables (p-value=0), processed meats, poultry, and fish (p-value=0), other dairy products (p-value=0), sweet snacks (p-value=0.001), sugared beverages (p-value=0), fats and oils (p-value=0.001), respectively. The FCS decreased by 4.6%. As food-related behaviors, most cooking attitudes, and practices (10 out of 13) showed an amelioration during the lockdown and the proportions of food stocked have been changing since the start of the pandemic seeing higher amounts of pasta, rice or other grains, flour, and legumes/pulses stocked.

**Conclusion:** To conclude, the hostile home isolation strategy followed to prevent the COVID-19 spread in Lebanon, came at a high nutritional cost, driving poor dietary diversity.

## Introduction

The food deprivation and acute hunger faced by 25.9% of the global population and around 67 million people across all countries, make the public health importance of food consumption patterns and food security indisputable. ^1^^,^^2^ In conflict-affected countries, disruption in agriculture and trade lead to the increase in the price of a simple plate of food that can cost more a day’s wages. ^2^ Nowadays, almost two years after the first case was discovered, the SARS-CoV-2 (COVID-19) virus has affected more than 220 million people in 188 countries, more than 14 million people in the Eastern Mediterranean Region and caused more than four million deaths globally and around 260 million people died in the Eastern Mediterranean area. ^3^ In Lebanon, from January until September 2021, there have been more than 700,000 confirmed cases of COVID-19 with around 8000 deaths, reported to the World Health Organization (WHO). ^4^

As a consequence of the pandemic, the loss of livelihoods and remittances led to food supply disruptions and lack of income limiting the access of households to nutritious food, mainly at poorer and vulnerable population levels. ^1^ Furthermore, around 1.5 billion people were not able to afford a nutrient-dense diet in which essential nutritional requirements are met. ^1^^,^^2^^,^^5^ At the regional level, Eastern Mediterranean countries are faced with scarce and dwindling natural resources amidst high urbanization rates, population increases, wars, climate change, sociopolitical crises ^6^ and recently, the COVID-19 pandemic. ^7^ Lebanon, a small, densely populated country in the heart of a conflict-torn region, is experiencing a severe economic crisis in addition to the protracted Syrian refugee crisis. Along with the already-struggling economy, the unexpected COVID-19 pandemic has had disastrous sequels for all population groups in Lebanon, including food and medicine shortages, business closures, unemployment, and a significant drop in wages in a country where the national currency has lost more than 90% of its value. ^8^ Therefore, more Lebanese families are being pushed further into poverty due to a lack of urgent economic reforms, with nearly three million people in Lebanon in need of financial and social assistance. ^8^ The price of the basic food basket jumped by 340 percent, worsening food insecurity in Lebanon, with most Lebanese households fearful they may run out of food. ^8^ This is clearly emphasizing the need for a situational analysis of the different dimensions of food consumption patterns and diet diversity in Lebanon.

The lack of studies concerning the changes in food consumption, mitigation measures, and food challenges had made this issue all the more important. Thus, the purpose of this study is to examine the impact of COVID-19 pandemic on the food consumption patterns and the diet diversity indicators mainly the food consumption score (FCS) of people residing in Lebanon. This report analyzed data from a study by De Backer, C. *et al.* ^9^ but had a much more in-depth focus on data specific to Lebanon allowing for much more detailed and specific conclusions.

## Methods

Full details of the methods used across all of the countries involved can be found in the original cross-sectional study described by De Backer, C. *et al.* ^9^ Below the method used in Lebanon by the Lebanese team is outlined.

The online survey consisted of a cross-sectional study that was conducted in 38 different countries. Data related to inhabitants of Lebanon that have participated in this survey has been selected for the sake of analysis in this study. Questions of the survey were available in native Arabic language as well as other languages extending choices for the respondents. The survey was kept open between April 17th and June 25th and consisted of multiple blocks of information of which only a few variables are used and reported in this paper. Eligible participants included in this study were aged 18 years or older and of both genders residing in Lebanon during the COVID-19 crisis. Convenience sampling was used to recruit respondents and the survey was advertised via different social network platforms (Facebook, Instagram, Twitter, WhatsApp) as well as academic networks. The survey was managed and collected by a team in Lebanon including Maha Hoteit, Hussein Mortada, Rania Mansour and Elissa Naim (see acknowledgment). The questionnaire consisted of a validated online survey to collect information related to different topics including: sociodemographic and economic information, lockdown measures, cooking attitudes, shopping, food stock, and food frequency consumption in term of food portions per week (The question asked was: “how often did you eat the following [portions of] foods? Please indicate how often you consumed at least one portion of the following foods and drinks”). ^44^ Regarding questions related to cooking attitudes, shopping and food frequency consumption, respondents were asked to answer each question two times, indicating their behavior in both periods (before the COVID-19 pandemic and during the COVID-19 lockdown). FCS, which is a proxy indicator used to assess dietary diversity, was calculated using the frequency of consumption of different food groups by a household during the seven days before the survey. The calculation formula of the FCS is: (starches × 2) + (pulses × 3) + vegetables + fruit + (meat × 4) + (dairy products × 4) + (fats × 0.5) + (sugar × 0.5). ^18^ The FCS was calculated for each of the respondents based on their answers to the food frequency questionnaire. Prior to calculating the FCS score, response options were merged forming the following two categories: Less or equal to 4 times per week and more or equal to 5 times per week. Two different FCS were calculated, the first one was based on the answers of respondents about food frequency consumption before the lockdown and the second one was based on their answers during the lockdown. Everyone was then classified as having a high FCS (if it is greater than 42) or low FCS (less than 42). ^18^ To determine factors that may impact the FCS, a binary logistic regression was calculated. In this regression the FCS (high vs low) was the dependent variable. A backward approach was used and factors having a p-value<0.05 were considered significant. Odds ratio and its confidence interval were also calculated for each of the factors.

### Statistical analysis

Respondents’ characteristics classified as categorical variables were presented as frequencies (percentages) while means ± standard deviation (SD) were used for continuous variables. Different statistical tests were used: The chi-square test was used to investigate differences for categorical variables between groups (gender) while an independent t-test was applied for continuous variables. A p-value lower than 0.05 was considered significant. Statistical analysis was conducted on IBM SPSS Statistics (IBM Corp, SPSS statistics for Mac, Version 24. Armonk, New York) (RRID:SCR_019096).

### Ethical consideration

A consent form was attached at the beginning of the online survey and was required to be completed before continuing. The consent form protected participants by letting them know their rights and responsibilities while keeping their information confidential. Moreover, this study received the approval of the ethics committees at the University of Antwerpen (SHW_46) (the country leader of this project) and at all the concerned countries, including Lebanon (#CUER 22-2020) given that it was observational with respect of confidentiality and no traceability of respondents.

## Results

### Socio-demographic and economic characteristics

The survey was completed by a convenient sample of 2449 Lebanese participants. A total number of 167 participants were excluded from the study due to either: being aged below 18 years or not completing the survey. Thus, 2282 Lebanese participants were included in the present study. ^43^ Among them, 80.9% were females. Most participants were either adults (25 to 46 years old, 52.1%) or youth (19 to 20 years old, 37.7%). A very low percentage of adolescents (18 years) (8.4%) and elderly people (1.8%) had registered ( Table 1). The mean age of the respondents was 29.36 years with a SD of 12.221. Males who responded to this survey were significantly older than females who did (p-value=0) ( Table 1). In regard to the educational level, around 40.4% of the respondents had a bachelor’s degree when analyzing both genders together. In addition, a significant higher percentage (41.1%) of females had a bachelor’s degree compared with males (37.4%) (p-value=0) ( Table 1). The household composition was also analyzed before and during the lockdown in which most households were composed of three to five adults (more than 45%). This trend has been also observed in males and females each separately, except for female living in the same household before the lockdown in which the majority were composed of less than three female adults per household (47.1%) ( Table 1). When looking at economic characteristics, a similar percentage of respondents were students (40.5%) and active workers (40.9%) while the minority were unemployed (18.6%) before the COVID-19 lockdown. The percentage of unemployed individuals had increased to 32.6% during the lockdown. This increase has been also observed for males (8.7% before lockdown to 28.5% during lockdown) and females (20.9% before lockdown to 33.5% during lockdown). Moreover, the COVID-19 lockdown has also induced a loss of income among 62.9% of respondents. This loss of income was significantly higher (p-value=0.007) for men (68.6%) compared to women (61.6%). In addition, most respondents, when taken either all together (73.8%) or categorized as males (74.3%) and females (73.7%), struggle to make their wages last a month. And most even struggle to earn enough money for shopping (65.6%, 63.1% and 66.1% for all respondents, men, and women respectively) ( Table 1).

**Table 1.** Sociodemographic and socioeconomic characteristics of the studied population.

Variables	Overall N (%)	Male N (%)	Female N (%)	p-value
Age (mean ± SD)	29.36 ± 12.221	33.29 ± 15.184	28.43 ± 11.214	
Adolescents (18 years)	18	18	18	0
Youth	20.70 ± 1.346	20.51 ± 1.253	20.74 ± 1.361	0
Adult	35.99 ± 10.233	38.85 ± 11.382	35.24 ± 9.776	
Elderly	70.64 ± 6.427	70.41 ± 6.427	70.90 ± 6.585	
Age categories (%)				
Adolescents (18 years)	192 (8.4%)	30 (6.9%)	162 (8.8%)	0
Youth	860 (37.7%)	136 (31.2%)	724 (39.2%)	
Adult	1188(52.1%)	248 (56.9%)	940 (50.9%)	
Elderly	42(1.8%)	22 (5.0%)	20 (1.1%)	
Gender (%)				
Male	436 (19.1%)	NA	NA	
Female	1846 (80.9%)			
Education (%)				
Under a high school diploma	289 (12.7%)	65 (14.9%)	224 (12.1%)	0
High school diploma or equivalent	499 (21.9%)	99 (22.7%)	400 (21.7%)	
Bachelor's degree	922 (40.4%)	163 (37.4%)	759 (41.1%)	
Master's degree	487 (21.3%)	74 (17.0%)	413 (22.4%)	
Doctorate	85 (3.7%)	35 (8.0%)	50 (2.7%)	
Number of adults living in the same household before the lockdown (%)				
less than 3	360 (45.7 %)	51 (38.9%)	309 (47.1%)	0.179
3 to 5	369 (46.9%)	71 (54.2%)	298 (45.4%)	
More than 5	58 (7.4%)	9 (6.9%)	49 (7.5%)	
Number of adults living in the same household during the lockdown (%)				
less than 3	975 (43.3%)	200 (46.2%)	775 (42.6%)	0.403
3 to 5	1120 (49.8%)	204 (47.1%)	916 (50.4%)	
More than 5	156 (6.9%)	29 (6.7%)	127 (7.0%)	
Employment before the lockdown (%)				
Student	925 (40.5%)	134 (30.7%)	791 (42.9%)	0
Working	933 (40.9%)	264 (60.6%)	669 (36.2%)	
Didn't working	424 (18.6%)	38 (8.7%)	386 (20.9%)	
Employment during the lockdown (%)				
Student	819 (35.8%)	110 (25.2%)	709 (38.4%)	0
Working	720 (31.6%)	202 (46.3%)	518 (28.1%)	
Didn't working	743 (32.6%)	124 (28.5%)	619 (33.5%)	
Loss of income since lockdown (%)				
Yes	1436 (62.9%)	299 (68.6%)	1137(61.6%)	0.007
No	846 (37.1%)	137 (31.4%)	709 (38.4%)	
Struggle to make money last until the end of month (%)				
No	597 (26.2%)	112 (25.7%)	485 (26.3%)	0.802
Yes	1685 (73.8%)	324 (74.3%)	1361 (73.7%)	
Struggle to have enough money to shopping (%)				
No	786 (34.4%)	161 (36.9%)	625 (33.9%)	0.225
Yes	1496 (65.6%)	275 (63.1%)	1221 (66.1%)	

### Consumption of food groups and food consumption score

Tables 2 and 3 show the food groups consumption and the FCS in the overall population, in Lebanon.

**Table 2.** Food groups consumption in Lebanon, before and during COVID-19 pandemic.

		Lebanon		
		Before N (%)	During N (%)	p-value
Fruit (fresh or frozen)	4 or less	1178 (51.6%)	1300 (57%)	0
	5 or more	1104 (48.4%)	982 (43%)	
Vegetables (fresh or frozen)	4 or less	822 (36%)	979 (42.9%)	0
	5 or more	1460 (64%)	1303 (57.1%)	
Legumes/pulses	4 or less	1848 (81%)	1775 (77.8%)	0.001
	5 or more	434 (19%)	507 (22.2%)	
Nuts or nut spread (unsalted)	4 or less	1920 (84.1%)	1947 (85.3%)	0.147
	5 or more	362 (15.9%)	335 (14.7%)	
Processed meat/poultry/fish/vegetarian alternatives	4 or less	1857 (81.4%)	1991 (87.2%)	0
	5 or more	425 (18.6%)	291 (12.8%)	
Unprocessed fish	4 or less	2152 (94.3%)	2140 (93.8%)	0.366
	5 or more	130 (5.7%)	142 (6.2%)	
Unprocessed poultry	4 or less	2032 (89%)	2033 (89.1%)	1
	5 or more	250 (11%)	249 (10.9%)	
Unprocessed red meat *	4 or less	2043 (89.5%)	2041 (89.4%)	0.948
	5 or more	239 (10.5%)	241 (10.6%)	
Wholewheat bread, pasta, grains	4 or less	1757 (77%)	1694 (74.2%)	0.003
	5 or more	525 (23%)	588 (25.8%)	
White bread, pasta, grains	4 or less	1491 (65.3%)	1484 (65%)	0.79
	5 or more	791 (34.7%)	798 (35%)	
Milk	4 or less	1678 (73.5%)	1693 (74.2%)	0.433
	5 or more	604 (26.5%)	589 (25.8%)	
Other dairy products	4 or less	1039 (45.5%)	1155 (50.6%)	0
	5 or more	1243 (54.5%)	1127 (49.4%)	
Sweet snacks	4 or less	1664 (72.9%)	1734 (76%)	0.001
	5 or more	618 (27.1%)	548 (24%)	
Sugared beverages	4 or less	1282 (56.2%)	1359 (59.6%)	0
	5 or more	1000 (43.8%)	923 (40.4%)	
Fats and oils	4 or less	1814 (79.5%)	1878 (82.3%)	0.001
	5 or more	468 (20.5%)	404 (17.7%)	

* Unprocessed meats: (refers to all mammalian muscle meat including beef, veal, pork, lamb, mutton, horse, and goat).

**Table 3.** Food consumption score of Lebanese population before and during the COVID-19 pandemic.

Main outcome		Lebanon N (%)
FCS before the COVID-19 pandemic		93.9±41.9
FCS during the COVID-19 pandemic		88.1±44.4
*p*-value (before vs during)		0
FCS before the COVID-19 pandemic	Low	211 (9.2%)
	High	2071 (90.8%)
FCS during the COVID-19 pandemic	Low	315 (13.8%)
	High	1967 (86.2%)
Percentage of decline in the non-acceptable FCS		4.6%
p-value		0

*Fruits group*

Regarding the consumption of fruits (fresh or frozen), 57% of the Lebanese population consumed fruits equals or less than 4 times per week during the lockdown, compared to 51.6% before the lockdown (p-value=0). It was also observed that the percentage of people consuming fruits five times and more per week, was relatively higher before the lockdown (48.4%) compared to the consumption during the lockdown (43%; p-value=0) ( Table 2).

*Vegetables group*

As seen in Table 2, it appears that 64% of the population had higher consumption of vegetables (more than five times per week) before the lockdown compared to 57.1% during the lockdown (p-value=0). This statistic lead to the increase in the number of people in the four times or less category observed during the lockdown (42.9% vs 36% before the lockdown; p-value=0).

*Legumes and pulses group*

81% of people living in Lebanon, from the current study, were consuming legumes and pulses equals or less than 4 times per week before the lockdown; this category saw a decrease during the lockdown (77.8%). Consequently, the percentage of people frequently consuming legumes and pulses (five times and more per week) increased during the pandemic by 3.2% (22.2% during vs 19% before; p-value=0.001) ( Table 2).

*Nuts group*

As per Table 2, the percentage of people frequently consuming nuts and derivatives was higher before the lockdown (15.9% before vs 14.7% during), while, a slight decrease of 1.2% was observed during the lockdown; 85.3% of the population was consuming nuts and derivatives 4 times or less during the lockdown compared to before (84.1%). Yet, these results were not statistically significant (p-value=0.147).

*Processed meat/poultry/fish/vegetarian alternatives*

The frequency of consumption of processed meat/poultry/fish/vegetarian alternatives was higher (more than five times per week) before the pandemic (18.6% before vs 12.8% during; p-value=0). In addition, a decrease of 5.8% in the consumption of this food group (four times or less per week) was observed during the lockdown (87.2% during vs 81.4% before; p-value=0) ( Table 2).

*Unprocessed fish, unprocessed poultry, and unprocessed meats*

It was observed that the frequent consumption (five times and more per week) of unprocessed fish was higher during the pandemic (6.2% during vs 5.7% before) and less frequent consumption (equals to 4 times or less per week) was higher before the lockdown (94.3% before vs 93.8% during). Yet, the frequency of consumption of unprocessed poultry and meat remained unchanged before and during the lockdown ( Table 2). However, these results were not statistically significant (fish-p-value=0.366, poultry-p-value=1 and meat-p-value=0.948).

*White and whole wheat, bread, pasta, and grains*

The frequency of consumption of wholewheat bread, pasta and grains was higher during the lockdown (25.8% during vs 23% before), yet this consumption was lower before the lockdown; 77% of the Lebanese population was consuming this food group equals to four times or less before the lockdown compared to 74.2% during the lockdown (p-value=0.003). As for the frequency of consumption of white bread, pasta and grains, there was no significant differences before and during the pandemic (p-value=0.79) ( Table 2).

*Milk and dairy products group*

The consumption of dairy products was lower during the pandemic; 50.6% of the Lebanese population was consuming dairy products equals to four times and less during the lockdown vs 45.5% before. Moreover, we noticed a remarkable decrease during the lockdown, of 5.1% in the consumption of other dairy products (54.5% before vs 49.4% during; p-value=0.003). As for the milk consumption, no significant differences were stated before and during the pandemic (p-value=0.433) ( Table 2).

*Sugar group (products and beverages)*

The frequency of consumption of sugary beverages and products in the studied population was high before the lockdown; 27.1% and 43.8% of the population consumed sweet snacks (p-value=0.001) and sugary beverages (p-value=0) respectively, five time and more per week ( Table 2). However, a decrease of 3% in the consumption of this group was noted during the lockdown (snacks-p-value=0.001; beverages-p-value=0) ( Table 2).

*Fats and oils group*

A decrease by 3% in the consumption of fats and oils was observed during the lockdown (82.3% during vs 79.5% before; p-value=0.001). It was also observed that added fats and oils were more frequently consumed in the Lebanese population before the lockdown (20.5% before vs 17.7% during; p-value=0.001) ( Table 2).

*Calculation of the food consumption score*

The FCS was calculated based on the equation explained previously in the methods. Compared to the period preceding the pandemic, the mean levels and standard deviation (SD) of the FCS in Lebanon was equal to 93.9±41.9 and it decreased to 88.1±44.4 during the pandemic (p-value=0). Additionally, the percentage of people having low FCS has increased during the lockdown from 9.2% to 13.8% (p-value=0). These results are presented in Table 3.

### Food consumption patterns and food-related behaviors

*Cooking practices and barriers*

Table 4 shows that most cooking attitudes and practices (10 out of 13) showed a significant increase in the intra-COVID-19 pandemic. The highest increase was recorded for the response “not having access to foods/ingredients needed or wanted” (36.2% positive answers before lockdown compared to 51.1% during lockdown) (p-value=0). The only practices that showed a significant decrease during lockdown was “throwing away food leftovers” (30.4% before lockdown compared to 26.6% during lockdown) (p-value=0). Yet, a slight decrease was stated during the lockdown, in the attitude of “feeling confident about managing money to buy healthy food” and “feeling confident about cooking a variety of healthy meals” of 0.8% and 0.3% (p-value=0.474 and p-value=0.706) respectively. Moreover, it was observed that the attitudes of “don’t having the funds for the foods/ingredients needed or wanted” and “don't having the access to the facilities needed to cook or bake such as stove, oven, kitchen equipment” increased by 11% and 9.6% during the lockdown (p-value=0 and p-value=0), respectively, despite the null change in the practice of “cooking meals using healthy ingredients” (p-value=0.611). Similarly, a significant increase of around 8.7% and 6.7% was observed in the practice of “cooking with leftover food” and “using other parts of food label to make food choices” (p-value=0 and p-value=0), respectively. More than 66% of people were “changing recipes to make them healthier”, were “thinking about healthy choices when deciding what to eat “and were “planning meals to have a varied diet”. Additionally, 52.9% of people were “using the nutritional information panel (nutritional breakdown of the products) to make food choices “( Table 4).

**Table 4.** Food-related behaviors (cooking practices and barriers).

	Before N (%)		During N (%)		Variation (%)	p-value
	No	Yes	No	Yes		
Plan meals to include all food groups (A varied diet)	816 (35.8%)	1466 (64.2%)	767 (33.6%)	1515 (66.4%)	2.2	0.043
Think about healthy choices when deciding what to eat	720 (31.6%)	1562 (68.4%)	675 (29.6%)	1607 (70.4%)	2	0.054
Feel confident about managing money to buy healthy food	692 (30.3%)	1590 (69.7%)	710 (31.1%)	1572 (68.9%)	-0.8	0.474
Use the nutritional information panel (nutritional breakdown of the products) to make food choices	1136 (49.8%)	1146 (50.2%)	1074 (47.1%)	1208 (52.9%)	2.7	0.014
Use other parts of food label to make food choices	1143 (50.1%)	1139 (49.9%)	987 (43.3%)	1295 (56.7%)	6.8	0
Cook meals at home using healthy ingredients	487 (21.3%)	1795 (78.7%)	476 (20.9%)	1806 (79.1%)	0.4	0.611
Feel confident about cooking a variety of healthy meals	488 (21.4%)	1794 (78.6%)	496 (21.7%)	1786 (78.3%)	-0.3	0.706
Change recipes to make them healthier	774 (33.9%)	1508 (66.1%)	675 (29.6%)	1607 (70.4%)	4.3	0
Cook with leftover food	932 (40.8%)	1350 (59.2%)	732 (32.1%)	1550 (67.9%)	8.7	0
Throw away (leftover) food	1588 (69.6%)	694 (30.4%)	1676 (73.4%)	606 (26.6%)	-3.8	0
Money (I didn't have the funds for the foods/ingredients I needed or wanted)	504 (57.1%)	378 (42.9%)	407 (46.1%)	475 (53.9%)	11	0
Access to food (I didn't have access to foods/ingredients I needed or wanted)	563 (63.8%)	319 (36.2%)	431 (48.9%)	451 (51.1%)	14.9	0
Access to cooking facilities (I didn't have (access to) the facilities needed to cook or bake: stove, oven, kitchen equipment)	636 (72.1%)	246 (27.9%)	551 (62.5%)	331 (37.5%)	9.6	0

*Criteria for recipe selection*

With regards to the criteria for recipes selection, the lockdown was found to significantly increase the percentage of agreeing responses for all criterions. For instance, during the lockdown, more than 70% of people selected their recipes had few ingredients (73.7%) that are easily available at home (81%) or at store (79.8%), inexpensive (71.8%) or at good price (81.6%), and healthy (77.5%) ( Table 5). Before the pandemic, more than half to three quarters of the people living in Lebanon, agreed that they were selecting recipes that were achievable with few ingredients, were available at home or can be easily found at the store, inexpensive to prepare, healthy and cheap. During the lockdown, an increase ranging between 2.3% and 13.1% was observed in these patterns. The selection of recipes that were based on ingredients easily available at the store, was the only decrease and saw a decrease of 1.2% ( Table 5).

**Table 5.** Criteria for recipes selection before and during the pandemic in Lebanon.

Selection of recipes	Before N (%)			During N (%)			p-value	Variation (%)	
	Disagree	Neutral	Agree	Disagree	Neutral	Agree		Disagree	Agree
Achievable with few ingredients	174 (19.7%)	174 (19.7%)	534 (60.6%)	112 (12.7%)	120 (13.6%)	650 (73.7%)	0	-7	13.1
Achievable with the ingredients I have at home	98 (11.1%)	108 (12.2%)	676 (76.7%)	77 (8.7%)	91 (10.3%)	714 (81%)	0.002	-2.4	4.3
Achievable with ingredients that can be easily found at the store	77 (8.7%)	91 (10.3%)	714 (81%)	91 (10.3%)	87 (9.9%)	704 (79.8%)	0.208	1.6	-1.2
Inexpensive to make	147 (16.7%)	171 (19.4%)	564 (63.9%)	125 (14.1%)	124 (14.1%)	633 (71.8%)	0	-2.6	7.9
Healthy	89 (10.1%)	130 (14.7%)	663 (75.2%)	69 (7.8%)	130 (14.7%)	683 (77.5%)	0.027	-2.3	2.3
Good (cheapest) prices	91 (11.3%)	97 (12%)	618 (76.7%)	82 (10.2%)	66 (8.2%)	658 (81.6%)	0.008	-1.1	4.9

*Shopping*

Shopping practices have also been affected during the lockdown in Lebanon. Indeed, more than 76% of respondents agreed that they search more for cheapest prices before and during the lockdown ( Table 5).

During the lockdown, respondents showed a significant reduction of 7.3% less going physically to select and buy food, and that they preferred to order their food products online (variation of 3.6%) they also preferred having it delivered to their home rather than being delivered at a seller’s point (4.1%) (p-value=0.008). Regarding places of groceries shopping, there was a significant decrease during the lockdown for all the places except for shopping straight from the farmer/producer. In addition, it is remarkable that respondents had shown a disinterest in buying food at organic/fair trade shops or specialty stores during lockdown (p-value=0) ( Table 6).

**Table 6.** Dietary shopping practices before and during the pandemic in Lebanon.

		Before N (%)		During N (%)		Variation (%)	p-value
		No	Yes	No	Yes		
Organization of groceries shopping	I physically go to the supermarket, shop, market, farmer, vendor to select and buy food.	79 (9.8%)	727 (90.2%)	138 (17.1%)	668 (82.9%)	-7.3	0
	I order my food online and pick it up at a seller's point.	651 (80.8%)	155 (19.2%)	622 (77.2%)	184 (22.8%)	3.6	0.008
	I order my food online and have it delivered at home.	581 (72.1%)	225 (27.9%)	548 (68%)	258 (32%)	4.1	0.008
Places of groceries shopping	A supermarket	66 (8.2%)	740 (91.8%)	136 (16.9%)	670 (83.1%)	-8.7	0
	Corner store/convenience store	96 (11.9%)	710 (88.1%)	144 (17.9%)	662 (82.1%)	-6	0
	Organic/Fairtrade food shop	426 (52.9%)	380 (47.1%)	526 (65.3%)	280 (34.7%)	-12.4	0
	Straight from the farmer/producer	545 (67.6%)	261 (32.4%)	526 (65.3%)	280 (34.7%)	2.3	0.132
	Specialty stores: bakery, butcher, delicacy shop	177 (22%)	629 (78%)	290 (36%)	516 (64%)	-14	0
	Via meal kits/meal boxes (with all you need to cook a meal)	580 (72%)	226 (28%)	605 (75.1%)	201 (24.9%)	-3.1	0.033

*Food stock*

**Figure 1.**
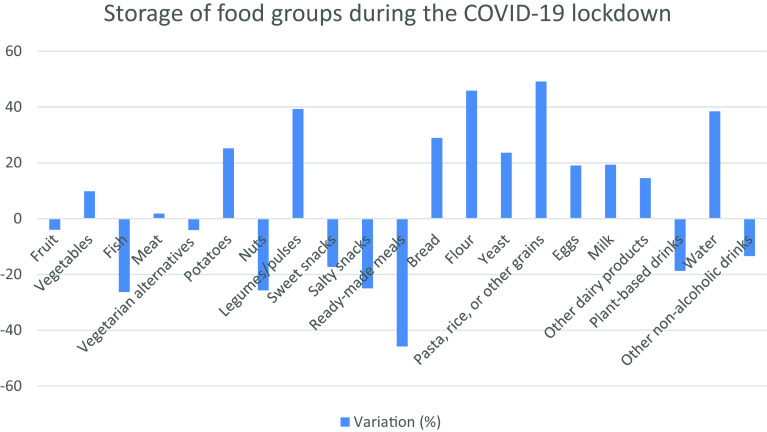
Food stock patterns during the pandemic in Lebanon.

Stocking up foods was also affected during the pandemic. Thereby, we observed a huge increase in stocking up pasta, rice or other grains, for flour, and for legumes/pulses (such as beans, lentils, chickpeas: dried or tinned). This increase was less significant for water, potatoes, yeast, milk, bread, vegetables, and meat. On the other hand, ready-made meals, salty snacks, nuts or nut spread, fish (fresh, frozen and canned), sweet snacks, and plant-based drinks were stored in smaller amounts during the lockdown. Furthermore, lockdown did not show any impact on stocking up non-alcoholic drinks, eggs, other dairy products, fruits in any of its forms as well as vegetarian alternatives ( Figure 1, Table 7).

**Table 7.** Food stock patterns during the pandemic in Lebanon.

		During N (%)			Variation (%)
		Less	Same	More	More-less
Food stock	Fruit (fresh, frozen, canned)	274 (34%)	290 (36%)	242 (30%)	-4
	Vegetables (fresh, frozen, canned)	227 (28.2%)	273 (33.8%)	306 (38%)	9.8
	Fish (fresh, frozen, canned)	369 (45.8%)	280 (34.7%)	157 (19.5%)	-26.3
	Meat (fresh, frozen, canned)	282 (35%)	227 (28.2%)	297 (36.8%)	1.8
	Vegetarian alternatives (fresh, frozen, canned)	279 (34.6%)	281 (34.9%)	246 (30.5%)	-4.1
	Potatoes	156 (19.4%)	290 (36%)	360 (44.6%)	25.2
	Nuts or nut spread (unsalted)	373 (46.3%)	267 (33.1%)	166 (20.6%)	-25.7
	Legumes/pulses (e.g., beans, lentils, chickpeas: dried or tinned)	130 (16.1%)	230 (28.5%)	446 (55.4%)	39.3
	Sweet snacks (e.g., sweets, cookies, pies, cakes)	342 (42.4%)	262 (32.5%)	202 (25.1%)	-17.3
	Salty snacks (e.g., crisps, salted nuts)	379 (47%)	250 (31%)	177 (22%)	-25
	Ready-made meals	472 (58.5%)	232 (28.8%)	102 (12.7%)	-45.8
	Bread	116 (14.4%)	341 (42.3%)	349 (43.3%)	28.9
	Flour	128 (15.9%)	180 (22.3%)	498 (61.8%)	45.9
	Yeast	164 (20.4%)	287 (35.6%)	355 (44%)	23.6
	Pasta, rice, couscous, or other grains	115 (14.3%)	180 (22.3%)	511 (63.4%)	49.1
	Eggs	154 (19.1%)	345 (42.8%)	307 (38.1%)	19
	Milk	197 (24.4%)	257 (31.9%)	352 (43.7%)	19.3
	Other dairy products (e.g., yoghurt, cheese)	185 (23%)	319 (39.5%)	302 (37.5%)	14.5
	Plant-based drinks (e.g., rice, oat, soy, almond)	320 (39.7%)	317 (39.3%)	169 (21%)	-18.7
	Water	80 (9.9%)	337 (41.8%)	389 (48.3%)	38.4
	Other non-alcoholic drinks	278 (34.5%)	358 (44.4%)	170 (21.1%)	-13.4

*Determinants of food insecurity in the overall population*

Many factors affected the FCS of the people living in Lebanon. Before the pandemic, the percentage of people having low FCS was 9.2% and increased to reach 13.8% during the pandemic ( Table 3). The determinants of poor dietary diversity indicated by “low FCS” in the overall population, before and during the pandemic, is conditioned by many variables including the gender, age categories, some cooking practices, cooking with leftovers, education, and employment. Prior to the pandemic, and compared to adolescents (18 years), elderly people were 86% less likely to be affected by the decrease in the FCS [OR:0.14; 95% CI (0.01-1.07)]. The cooking practice that affected the FCS of the population studied the most, was meal planning. 55% of people who planned their meals to include a variety of food [OR:0.45; 95% CI (0.31-0.65)] were less affected by the decrease of the FCS compared to those not practicing this pattern. As for the education, it appears that people with a high school diploma [0.3; 95%CI (0.11-0.82)] were 70% less likely to be affected by the decline in the FCS compared to those who attained lower levels of education. During the pandemic, the binary logistic regression analysis showed that the decrease in the FCS doesn’t seriously affect the elderly [OR: 0.17; 95%CI (0.04-0.72)], when compared with adolescents. Similarly, the decrease in the FCS didn’t affect people with a high school diploma [OR:0.26; 95% CI (0.1-0.66)] and a bachelor degree [OR:0.37; 95% CI (0.15-0.92)], when compared to people with a lower educational level. It was noticed that there was no impact of gender, cooking practices (except for planning meals to include a variety of food before the pandemic), cooking or throwing away leftovers, and employment on the FCS, neither before nor during the lockdown ( Table 8).

**Table 8.** Backwards Odds ratios (OR) according to food consumption score.

Independent variable	Binary logistic regression		p-value
Before lockdown	Odds ratio	OR Confidence Interval	
Gender: Female vs Male	0.815	[0.547-1.214]	0.315
Age group: Youth vs Adolescents	0.279	[0.033-2.348]	0.24
Age group: Adults vs Adolescents	0.186	[0.024-1.455]	0.109
Age group: Elderly vs Adolescents	0.143	[0.019-1.075]	0.059
Plan meals to include all food groups: positive vs negative	0.457	[0.318-0.658]	0
Think about healthy choices when deciding what to eat: positive vs negative	0.883	[0.594-1.314]	0.54
Feel confident about managing money to buy healthy food: positive vs negative	1.44	[0.982-2.112]	0.062
Use the nutritional information panel: positive vs negative	1.207	[0.826-1.763]	0.33
Use other parts of food label to make food choices: positive vs negative	0.797	[0.549-1.157]	0.233
Cook meals at home using healthy ingredients: positive vs negative	1.008	[0.628-1.617]	0.974
Feel confident about cooking a variety of healthy meals: positive vs negative	0.853	[0.524-1.390]	0.524
Change recipes to make them healthier: positive vs negative	0.997	[0.678-1.466]	0.988
Cook with leftover food: positive vs negative	0.941	[0.687-1.290]	0.706
Throw away leftover food: positive vs negative	0.94	[0.680-1.300]	0.709
Education level: High school diploma vs under a high school diploma	0.304	[0.113-0.821]	0.019
Education level: Bachelor's degree vs under a high school diploma	0.547	[0.204-1.469]	0.232
Education level: Master's degree vs under a high school diploma	0.604	[0.232-1.571]	0.301
Education level: Doctorate vs under a high school diploma	1.249	[0.456-3.424]	0.665
Employment status: I worked vs I was a student	1.096	[0.628-1.912]	0.748
Employment status: I didn't work vs I was a student	1.046	[0.690-1.586]	0.833

During lockdown	Odds ratio	OR Confidence Interval	p-value
Gender: Female vs Male	0.794	[0.567-1.112]	0.18
Age group: Youth vs Adolescents	0.365	[0.076-1.749]	0.208
Age group: Adults vs Adolescents	0.255	[0.057-1.137]	0.073
Age group: Elderly vs Adolescents	0.17	[0.040-0.728]	0.017
Plan meals to include all food groups: positive vs negative	0.87	[0.616-1.229]	0.428
Think about healthy choices when deciding what to eat: positive vs negative	0.863	[0.600-1.242]	0.428
Feel confident about managing money to buy healthy food: positive vs negative	1.021	[0.735-1.417]	0.902
Use the nutritional information panel: positive vs negative	0.989	[0.685-1.426]	0.951
Use other parts of food label to make food choices: positive vs negative	0.829	[0.575-1.195]	0.315
Cook meals at home using healthy ingredients: positive vs negative	0.742	[0.487-1.129]	0.163
Feel confident about cooking a variety of healthy meals: positive vs negative	0.902	[0.590-1.379]	0.633
Change recipes to make them healthier: positive vs negative	1.13	[0.794-1.608]	0.497
Cook with leftover food: positive vs negative	1.102	[0.833-1.459]	0.496
Throw away leftover food: positive vs negative	0.887	[0.667-1.180]	0.411
Education level: High school diploma vs under a high school diploma	0.267	[0.108-0.661]	0.004
Education level: Bachelor's degree vs under a high school diploma	0.379	[0.155-0.924]	0.033
Education level: Master's degree vs under a high school diploma	0.453	[0.190-1.082]	0.075
Education level: Doctorate vs under a high school diploma	0.853	[0.347-2.095]	0.728
Employment status: I worked vs I was a student	0.978	[0.610-1.566]	0.925
Employment status: I didn't work vs I was a student	1.045	[0.734-1.487]	0.807

## Discussion

The present study aimed to investigate the changes in food consumption patterns, diet diversity through the assessment of the FCS and food-related behaviors in Lebanon during the lockdown. In the pre-pandemic and intra-pandemic periods, most food groups were consumed in a frequency of equals to four times and less per week. One in two people were consuming fruits and two in five people were consuming vegetables. Moreover, four out of five people were consuming legumes and pulses and four in five people were consuming nuts.

As for unprocessed fish, poultry, and meats, nine in ten people were consuming these foods and three out of four people were consuming wholewheat bread, pasta, and grains. The consumption of milk and other dairy products were shown in three out of five people, and four in five people were consuming added fats and oils. On the other hand, some food groups were consumed frequently (more than five times per week): 4 in 25 people were consuming processed meat/poultry/fish and vegetarian alternatives, 17 in 50 people consumed sugary products, and 7 out of 20 were consuming refined grains group. The confinement induced an increase in the consumption of legumes and pulses (3.2%, p-value=0.001) and whole wheat groups (2.8%, p-value=0.03). In contrast, a decrease of 5.4%, 6.9%, 5.8%, 5.1%, 3.1%, 3.4% and 2.8% was observed in the consumption of fruits (p-value=0), vegetables (p-value=0), processed meats, poultry, and fish (p-value=0), other dairy products (p-value=0), sweet snacks (p-value=0.001), sugared beverages (p-value=0), fats and oils (p-value=0.001), respectively. The average FCS decreased by 4.6% during the lockdown. Most cooking practices (10 out of 13) showed a significant increase during the lockdown and the proportions of food stocked have been changing since the start of COVID-19 with larger amounts of pasta, rice or other grains, flour, and legumes/pulses being stocked. No such change was observed in the storage of non-alcoholic drinks, eggs, other dairy products, fruits in any of its forms, as well as vegetarian alternatives. Since 2020, Lebanon is facing economic and political turmoil that has no sign of ending presently. In addition, the country is densely populated with 6.9 million people, 87.2% of whom live in urban areas, including 2 million displaced persons and 500,000 migrant workers. ^9^ Households are primarily multi-generational households, with an average of five people per household. ^10^ Several additional challenges were observed throughout the pandemic, such as the absence of food, having to travel to several places to find it, inability to afford some food types, worries on food safety, and finding the best-price shops for buying some foods.

The preventive measures for COVID-19 (i.e., physical distancing) and the international recommendations concerning restrictions on travel that were imposed by WHO and Lebanese government, along with the fear of having COVID-19 infection, have led to quarantining of millions of people. This has meant that the tourism, the hospitality industries, the food supply chains, the health system and the industry sector have all seen a sharp decline. Moreover, the production and transportation of fresh nutritious food, such as fruits and vegetables, meat, milk, and other dairy products were affected during the pandemic. In most countries, the cheap highly processed, packaged foods characterized by long shelf life, highest amounts of total fats, saturated fats, sugars and/or salt were frequently consumed leading to lower quality diets. ^1^ Upon the exponential increase in COVID-19, with the consequences on the financial status of consumers, food insecurity started to aggravate. Consumers tend to limit the purchase of food types they cannot afford such as meat and fish and start consuming higher quantities of starchy food due to their wide availability and cheap prices. ^11^

Food consumption and dependency on specific food groups may also be related to the curfew consequences of this virus. In other words, the mental status and anxiety related to food availability can push food insecure consumers toward the consumption of more fruits, savory snacks, sweets, and candies which in turn can cause weight gain. ^12^ Furthermore, the COVID-19 pandemic was acquainted by a need for food-based and non-food based coping strategies to compensate for household economic crisis. Internationally, all sources of income were affected which led to a reduction in resources and an increase in food insecurity prevalence rates. ^1^ Consumers were forced to change their food consumption patterns involuntarily, rely on savings, sell durable household assets and livestock, buy foods with a long shelf-life, eat less, buy cheaper food, and accept food from a friend as a mean of food-based coping. Regarding non-food based coping strategies, consumers relied more on savings, obtained credits, sent household members elsewhere, signed up for governmental programs and borrowed money from friends or family. ^13^ Still remaining was the impact of COVID-19 on the access and availability of resources which constitutes the base to start combating food insecurity.

This pandemic has altered the agriculture sector in Lebanon, food demand, hunger and malnutrition, and world food prices that exacerbated the food security status. ^14^ The FCS is used to assess the dietary diversity and relative food frequency along with the household’s food security. The decrease in the FCS in Lebanon may be related to several factors such as the critical political and economic situations affecting the country. ^15^ Clashes, political conflicts, and civil insecurity are the main driving factor of food insecurity in Lebanon in recent years due to numerous factors such as refugee migration, army conflicts and political instability. ^6^ In this study, other factors were influencing the FCS such as planning meals, education level, and age category. In fact, people who planned their meals and gave many concerns to variety foods, people with a high school diploma and bachelor’s degree, and elderly people were less affected by the decline of FCS. All these factors may be directly related to meal organization abilities, knowledge, economic status, and person responsible for preparing food.

To mitigate the effects of income losses and decrease in purchasing power, people started to adjust to these shortages through different coping strategies. Among these strategies, a significant change in most cooking practices was observed such as cooking more with leftovers in addition to minimizing food waste. Furthermore, COVID-19 had led people suffer a significant increase in barriers to cooking healthy meals due to the unavailability of money and less accessibility to food and cooking facilities. During the curfew, people searched more for inexpensive recipes that could be achieved with fewer ingredients. This can be explained by the financial struggle communities are having. These struggles also lead to more people preserving food leftovers rather than throwing them away and ensuring enough resources for healthy cooking with a high emphasis on the cultural role of females in cooking. ^16^ Even when purchasing ingredients, people tend to search more for cheaper prices and less organic foods but order them online rather than going physically to the stores. ^13^^,^^17^^–^^20^ These statements came hand in hand with our findings where the most cooking practices (10 out of 13) showed a significant increase during the lockdown. The highest increase was recorded for not having access to foods/ingredients needed or wanted and the lowest one for throwing away food leftovers. Besides, proportions of food stocked have been changing since the start of COVID-19 and higher amounts of pasta, rice or other grains, flour, and legumes/pulses were stocked with an unchanged consumption rate of non-alcoholic drinks, eggs, other dairy products, fruits in any of its forms as well as vegetarian alternatives. This may be related to the perceived need for these food groups and their cheap prices.

### Comparison with other countries

When comparing our results with findings from other countries’ and studies, we observed that the home isolation due to the pandemic induced changes in food-related behaviors in Russia, of which a decrease of meat consumption and sweet products was observed in 1047 adults along with an adoption of healthier consumption patterns. ^21^ In addition, an American survey (n=484 adult participants), suggested people facing food insecurity reduced their intake of fruits and vegetables since the start of the pandemic. ^22^ The findings of the American study, as well as our findings, supported two Italian surveys. One of which stated 18% of the respondents reported a decrease in the consuming of fresh fruits while in another study, 8.7% of the respondents reported a decrease in the consumption of fresh fruits and vegetables. ^23^^,^^24^ However, a third study conducted in Italy stated that 2768 adults showed an improvement in their diet quality in which they increased their consumption of fruit (24.4%), vegetables (28.5%), legumes (22.1%), nuts (12%), and fish or shellfish (14%). On the other hand, an excessive consumption of sweets or pastries (36.9%) increased in this study. ^25^ A survey by Enriquez-Martinez *et al.*, conducted in Argentina, Brazil, Mexico, Peru, and Spain on 6,325 adults revealed that most Spanish participants (61.6%), didn’t show any improving or worsening in their food pattern. ^26^ People living in Argentina and Brazil reported some amelioration in their diets. Lower change in food consumption patterns was observed among Peruvians and Mexicans (OR: 0.51; 95% CI: 0.4–0.6 and OR: 0.69; 95% CI: 0.4–0.8, respectively), when compared to Argentinians. ^26^ A Polish survey enrolled in Poland (n=2381 adults) shows that 30% of respondents improved their food patterns through a rise in the consumption of vegetable, milk and other dairy products. In addition, the consumption of fast food, and commercial pastry along with confectionary and salty snacks decreased in more than 50% of respondents. Moreover, one-fifth of the respondents reduced the intake of sugar-sweetened beverages and alcohol. ^27^ In France, in comparison to the period preceding the pandemic, the nutritional quality of the respondents (n=938) diets was affected strongly; a sharp increase in processed meat, sweet-tasting beverages, and alcoholic beverage consumption had negatively decreased the overall diet quality. ^28^ In addition, a French web-based survey that encompassed 37,252 adults showed a decline in the intake of fresh food of where 17% of participants reported a decrease in consumption of fresh fruits, 18% reported a decrease in consumption of fresh vegetables, 22% in fresh red meats, and 31% in fresh fish. On the other hand, 22% of people reported an increase in the consumption of sweets and chocolate, 20% in cookies and cakes, and 18% in cheese along with a decline in the consumption of sandwiches, pizzas, or savory pies (17%). ^29^ The Lithuanian COVIDiet Study (n=2447 participants) that revealed frequent and high consumption of sweets, biscuits, cakes, frozen and canned foods and low intake of fruits and vegetables, also reported frequent cooking and eating out of control. However, this was associated with some positive changes such as high consumption of fresh fruits, vegetables, fish, legumes, and white meat and low consumption of processed meat, carbonated and sugary drinks. ^30^ The findings from a survey conducted by three European countries (n=1071 adults): from Poland (n=407), Austria (n=353) and the United Kingdom (n=311) showed a frequent purchase of frozen foods with long shelf life and high consumption of dairy products, grains, fats, vegetables, and sweets (p-value<0.05). ^31^ In Belgium, (n=8640 adults), 10.4% of participants reported food shortages and 10.3 % were incapable of eating a nutritious diet during lockdown. This status of food insecurity was associated with a change in most dietary behaviors. ^32^ The people living in Denmark, Germany, and Slovenia witnessed, during the pandemic, a decline in the intake of fresh food. ^33^ Three studies in Brazil were conducted between 2020 and 2021 in which more than 50,000 adults were interviewed through web-based questionnaires. The first study highlighted the increase in consumption of processed and energy dense foods (potato fries, chocolate, and ice cream) among Brazilian adults. ^34^ In the second study, an increase in the consumption of vegetables, fruits, and legumes along with a steadiness in the consumption of processed foods was observed. ^35^ The third study described the reduction in consumption of fruits and vegetables along with an increase in the consumption of candies and fast-food. ^36^ In Chile (n=700 adults), it was shown that there was a low consumption of legumes and water and high consumption of junk food (e.g., food with low food quality, low contribution of micronutrients and with a high contribution of sugar, saturated fat, and sodium) and fried foods. ^37^ In China (n=2702 adults), many participants didn’t witness any modification in their habitual diet, while 38.2% increased their snack intake, during their home isolation. This could be explained by the fact that basic food supplies were offered by the government in China during the lockdown. ^38^ Similarly, in the Netherlands, 83% of participants did not modify their eating habits during their confinement. ^39^ A scoping review on many populations (US, Asia including Palestine, India, and China, Europe including Italy, France, Spain, Poland, and the UK, Australia, and Zimbabwe) showed an increase in the consumption of meals and snacks, an amelioration in dietary patterns associated with an increase in fresh produce and home cooking and reductions in comfort food and alcohol consumption. However, in the same review, nine studies showed a reduction in fresh produce, with a further six describing an increase in the consumption of comfort foods such as sweets and processed foods. An increase in alcohol consumption was reported in two studies and weight gain was reported in fifteen studies a reduction in physical exercise. ^40^

### Comparison with other Eastern Mediterranean regional countries

At regional level, in Jordan, among a total of 3129 Jordanians, 23.1% of the respondents were facing severe food insecurity, 36.1% were moderately food insecure and 40.7% were food secure. Food insecure people were consuming fewer meats and carbohydrates compared to food secure people. ^11^ In Iran, the household’s dietary diversity and food security status were studied and a decline in the consumption of white roots and tubers, dark green leafy vegetables, other fruits, organ meat, legumes, nuts and seeds, sweets, spices, condiments, and beverages was shown. ^41^ In addition, the impact of COVID-19 pandemic on food purchasing and dietary behaviors was studied in three Kuwaiti surveys with 1935 respondents in Kuwait. In the first study, an increase in the consumption of vegetables, fruits, and carbohydrates along with a decrease in the consumption of fish and sugary drinks was observed. ^42^ In the second study, a change in the consumption of only fish and seafood was observed. ^17^ In the third study, 50% of respondents reported no changes at all in the consumption of all food groups (44%). In addition, more than half the participants ate more fruits and vegetables, 41.5% ate more legumes and pulses, and 2.3% ate fewer fast food. ^18^ In Lebanon, more than 35% of people did not eat fruits and vegetables daily. In addition, 28% consumed sweets or desserts and 24% drink sweet beverages at least once per day and 30.9% consumed salty snacks (nuts, crackers, chips). ^19^ Moreover, according to Hoteit *et al.*, 9 in every 16 households ate less than one or two meals daily and the majority of them were omitting their meals to spare food. In addition, more than 50% of the Lebanese population were having a low food consumption score. ^15^ Another study conducted in three countries of which Lebanon was included, showed that 33% of people were shopping for their food once per week and 31% of the respondents stated that they were shopping two to three times a week during the COVID-19 pandemic. One in every four were purchasing food less than once a week. Less than 10% of people living in Lebanon, Tunisia, and Jordan were obtaining food and groceries from supermarkets or food delivery services and was significantly the least reported among the Tunisians. ^20^

### Limitations

Some limitations should be considered when evaluating the results of this study. This is a cross-sectional study of which it included retrospective data on the time before lockdown. Thus, due to the respondents’ memory, the presented eating habits could be biased due to the type of questions asked concerning the food patterns before the pandemic. Another limitation concerns the determination of the portion size. Thus, it was not possible to use home measurements or the photographs of products to assist respondents in determining the food portion size.

## Conclusion

The results of the study indicated some changes in the food consumption patterns with a decline in the dietary diversity that took place due to the social isolation implemented during the COVID-19 pandemic. The reduction in the frequency of consumption of fruits, vegetables, processed meats, poultry, and fish, dairy products (e.g., cheese), sweet snacks, sugared beverages, and fats and oils was clearly observed in our population. On the other hand, an increase in the consumption of legumes and pulses, and whole wheat group was observed also. In the short term, according to our findings, nutrition behavior and the dietary diversity did change during lockdown. Nevertheless, most cooking practices showed a significant increase during the lockdown in the overall population and the proportions of food stocked have been changing since the start of the pandemic where higher amounts of pasta, rice or other grains, flour, and legumes/pulses were stocked with an unchanged storage rate of non-alcoholic drinks, eggs, dairy products, fruits, and vegetarian alternatives. All these changes in dietary behaviors might lead to a negative health consequence. It is essential to foster the governmental role along with the social programs, non-governmental agencies, and public health interventions in encouraging the revitalization of the Lebanese traditional healthy cuisine.

## Data availability

### Underlying data

OSF: CORONACOOKING COVID-19 Lebanon. https://doi.org/10.17605/OSF.IO/PRTDQ. ^43^

The project contains the following underlying data:

- Lebanon_DataSet.sav (owned by the corresponding authors).

### Extended data

OSF: Supplementary materials CoronaCookingSurvey April 2020 https://doi.org/10.17605/OSF.IO/8GPZX. ^44^

The project contains the following extended data:

- Corona_Cooking_Survey_English_Food security.pdf

Data are available under the terms of the Creative Commons Zero “No rights reserved” data waiver (CC0 1.0 Public domain dedication).
