# Supplementary material for: COVID-19 home isolation and food consumption patterns: Investigating the correlates of poor dietary diversity in Lebanon: a cross-sectional study
Source: F1000Res. 2022 Jan 28;11:110. [Version 1] doi: 10.12688/f1000research.75761.1 (PMC8864186; doi:10.12688/f1000research.75761.1)
Supplement: Peer review history

# Peer review: Reviewer response for version 1

**DOI:** 10.5256/f1000research.79679.r121391

**Published:** 2022-02-22

**Authors:** Sabika S. Allehdan

**Affiliations:** 1 Department of Nutrition and Food Technology, Faculty of Agriculture, The University of Jordan, Amman, Jordan

I appreciate the opportunity to review this interesting manuscript. I enjoyed reading the manuscript. This study aims to explore food consumption pattern and dietary diversity among Lebanese adults during COVID-19 lockdown. Overall, this is a high quality, clear, concise, and well-written manuscript. This manuscript has great implications for nutrition and public health policy. There are specific comments that should be clarified to improve the scientific quality of this manuscript.

 First: Clarify how you calculated the sample size of this study in the methods section.

 Second: Explain how you calculated ORs and their CIs as well as P-values in the statistical analysis section as you calculated them in table 8.

 Third: In table 1, the first five rows represent data as mean ± SD not as (number) (%). Where are the P-values of t-test and chi-square? It is obvious that mean age of females was significantly lower than mean age of males.

 Fourth: Please determine serving (servings or cups or ounces) and timing pattern (daily /weekly) of food groups consumption in table 2 (second column). For example, '4 or less' or '5 or less' servings, cups or ounces per day or week.

 Fifth: Food consumption score displayed in table 3 as mean ± SD not as (number) (%) in 2 and 3 rows, so change the name of the last column header in table 3.

 Sixth: Insert a legend for each table. Legends should include full name of abbreviation. For example, spell the full name of FCS in the legend of table 3. Identify name of statistical analysis was used to calculate P-value in legend for each table.

Is the work clearly and accurately presented and does it cite the current literature?

Yes

If applicable, is the statistical analysis and its interpretation appropriate?

Yes

Are all the source data underlying the results available to ensure full reproducibility?

Yes

Is the study design appropriate and is the work technically sound?

Yes

Are the conclusions drawn adequately supported by the results?

Yes

Are sufficient details of methods and analysis provided to allow replication by others?

Yes

Reviewer Expertise:

Clinical Nutrition  and epidemiological nutritional

I confirm that I have read this submission and believe that I have an appropriate level of expertise to confirm that it is of an acceptable scientific standard.

# Peer review: Reviewer response for version 1

**DOI:** 10.5256/f1000research.79679.r121394

**Published:** 2022-02-21

**Authors:** Nahla Al Bayyari

**Affiliations:** 1 Department of Nutrition and Food Technology, Faculty of Al-Huson University College, Al-Balqa Applied University, As-Salt, Jordan

The manuscript examine the impact of the COVID-19 pandemic on food consumption patterns and dietary diversity of the Lebanese people. The authors clearly and accurately presented and cited the current literature. The study design was a cross-sectional which is technically appropriate for this type of studies. Sufficient details of methods and analysis were provided and all statistical analysis methods are fit with the study design and variables of the study as well as all interpretations are correct. All data underlying the results available in tables are matched with the results part. The study conclusions were based on the study results which indicated some changes in the food consumption patterns with a decline in the dietary diversity that took place due to the social isolation implemented during the COVID-19 pandemic. 

Is the work clearly and accurately presented and does it cite the current literature?

Yes

If applicable, is the statistical analysis and its interpretation appropriate?

Yes

Are all the source data underlying the results available to ensure full reproducibility?

Yes

Is the study design appropriate and is the work technically sound?

Yes

Are the conclusions drawn adequately supported by the results?

Yes

Are sufficient details of methods and analysis provided to allow replication by others?

Yes

Reviewer Expertise:

Public health nutrition as well as clinical nutrition surveys and the randomized double blinded clinical trials.

I confirm that I have read this submission and believe that I have an appropriate level of expertise to confirm that it is of an acceptable scientific standard.
